# How UK internet websites portray breast milk expression and breast pumps: a qualitative study of content

**DOI:** 10.1186/s12884-015-0509-0

**Published:** 2015-04-02

**Authors:** Rhona J McInnes, Alix Arbuckle, Pat Hoddinott

**Affiliations:** School of Health Sciences, University of Stirling, Stirling, FK9 4LA UK; Master of Health Research (MRes) programme, School of Health Sciences, University of Stirling, Stirling, FK9 4LA UK; Nursing Midwifery and Allied Health Professions Research Unit, School of Health Sciences, University of Stirling, Stirling, FK9 4LA UK

**Keywords:** Health information, Internet, Breastfeeding, Breast pumps, Expressing milk, Infant feeding, Health inequalities, Bonding

## Abstract

**Background:**

Exclusive breastfeeding for six months is recommended but few parents achieve this; particularly younger and less well-educated mothers. Many parents introduce infant formula milk to manage feeding but describe a desire to express breastmilk alongside a lack of support or information. The Internet is highlighted as a key resource. This study aimed to examine UK websites on expressing breastmilk to identify key messages and how information is provided.

**Methods:**

We used search terms in Google to identify websites with information rich content on expressing breastmilk and breast pumps. Ten sites were purposively selected at two time points in 2013 and 2014 to represent 3 categories: commercial, NHS or 3^rd^ sector (voluntary or not for profit). Each site was reviewed by two researchers, data and reflective analytical notes were uploaded into NVivo and thematic data analysis undertaken.

**Results:**

Sites varied considerably in their design, use of images, videos, audio files, product placement and marketing opportunities. Three key themes emerged: depiction of expressing; reasons to express; and recommendations about expressing. Inconsistent and conflicting information was common within and between sites. Expressing was portrayed as similar to, but easier than, breastfeeding although at the same time difficult and requiring to be learned. Expressed breastmilk is promoted by mainly commercial sites as immediately available, although pumps were also presented as needing to be concealed, not heard or seen. Health benefits were the overarching reason for expressing. Although predicated on separation from the baby, commercial sites identified this as a positive choice while other sites focused on separation due to circumstance. Commercial sites emphasised restrictions related to breastfeeding, lack of sleep and bonding with the father and wider family. Non-commercial sites emphasised hand expression, with some not mentioning breast pumps. Practical information about starting expressing in relation to infant age or duration of breastfeeding was conflicting.

**Conclusions:**

Internet information about expressing breastmilk is inconsistent, incomplete and not evidence informed. The lack of research evidence on the relationship between expressing and feeding outcomes has provided opportunities for commercial companies, which have the potential to further exacerbate observed health inequalities. Access to good quality information based on robust evidence is urgently required.

## Background

Good quality evidence of the short and long term benefits of breastfeeding identifies it as ‘a major public health issue’ [1]. Infants who are not breastfed are at increased risk of poorer health outcomes and poorer neurological development [2]. A decreased incidence of breast and ovarian cancer in mothers is associated with accumulating more than 12 months of breastfeeding [2]. These studies have indicated that both duration and exclusivity of breastfeeding affect maternal and infant health outcomes [1,2] and underpin a global recommendation for countries to promote exclusive breastmilk feeding for the first six months of life [3]. Yet, in the UK less than 1% of babies are exclusively breastfed to six months and duration of breastfeeding has changed little in the last decade despite increases in breastfeeding initiation [4]. Data collected in Scotland [5] show an increase in mixed feeding and a slight decrease in exclusive breastfeeding in recent years. In the most recent UK infant feeding survey [4] 73% of women had introduced infant formula in the first six weeks of life and earlier introduction was associated with younger age and being in routine or manual occupations or unemployed.

Expressing breastmilk is important because it provides an alternative to breastfeeding which enables the baby to continue to be fed breastmilk exclusively when breastfeeding is either not preferred or desired. The other option is for families to introduce infant formula milk; however the use of formula milk feeding is associated with poorer health outcomes as discussed above. Although the relative health benefits of breast milk compared to breastfeeding are poorly understood, a RCT in premature infants showed lower levels of life threatening neonatal infections in infants fed expressed breast milk compared to those fed infant formula milk [6]. Furthermore combining breastfeeding with infant formula is associated with shorted breastfeeding duration [4].

Expressing breastmilk is increasing in prevalence according to recent studies from Australia and the USA [7,8]. There are three methods: hand expression (without the assistance of a pump); hand operated pumps and electric pumps, however very little is known about current practices. An estimated 85% of USA mothers of infants aged 1½ to 4½ months are expressing breastmilk, mainly using electric pumps [9]. An Australian study [10] identified that around 46% of mothers of healthy term infants were expressing breastmilk in the first 24–48 hours following birth and that 47.9% of primiparous women in their sample already had a breastpump before birth. The prevalence of expressing and pump use in the UK is unknown as neither of the main national databases (i.e. routine data collected by primary care health professionals such as Information and Statistics Division Scotland [5], Vital Signs Monitoring [11]) nor the self-report 5-yearly National survey data [4] ask about it. UK postnatal recommendations ([12] pg. 24) are that ‘all breastfeeding women should be shown how to hand express’, that ‘breast pumps should be available in hospital, particularly for women who have been separated from their babies’ and that ‘all women who use a breast pump should be offered instructions on how to use it’. However actual expressing practices are unknown.

We have identified two systematic reviews of expressing breastmilk [13,14]. Becker et al. [13] evaluated expressing in terms of acceptability, effectiveness, safety, effect on milk composition, contamination and costs but did not provide a definitive answer for the best method, indicating that this depended on age of infant, reasons for expressing and individual preference. Health outcomes or duration of breastfeeding were not specifically assessed. Nine of the 23 papers in the review reported on term infants and only one of these was from the UK. The other systematic review [14] focused on expressing breastmilk by mothers of healthy term infants, including prevalence, purpose, methods and outcomes. This review identified seven relevant papers (none of which were from the UK) and concluded that there was limited evidence on the prevalence and outcomes of expressing breast milk. The authors also stated that expressing breastmilk had increased as had the range of commercially available infant feeding equipment [14]. Our interview study with families [15] identified that attitudes to and experiences of expressing breastmilk were different from recommended practice [12] and that women and their families wanted more support for their decisions. Women in our study also identified internet websites as key information sources for making feeding decisions.

General concerns have been expressed over the quality and completeness of health information available on the Internet [16,17]. One study about infant colic [17] identified variable content and differences between commercial and non-commercial websites, which may expose parents to conflicting or potentially harmful information. Most of the research literature on Internet provision of health information has focused on the quality and quantity of available information or on delivering health interventions via the Internet or website applications [18]. Few qualitative studies have investigated the social and cultural meanings of the available information. The aim of this study was to examine websites that women might access when seeking information about expressing their breastmilk, to identify key messages and how information is provided.

## Methods

### Identification of websites

A search strategy using Google, a popular search engine that mothers would be likely to use, to search for the terms ‘expressing’, ‘expressing breastmilk’, ‘help with expressing’, ‘pumping breastmilk’, ‘breast pumps’. These terms were agreed by RM and AA and validated by exploring service users’ entries on Mumsnet and from our knowledge of the 3^rd^ sector (voluntary or not for profit sector) and feeding resources available to women. This was not a formal academic literature search and the longer terms used were required to improve specificity.

### Inclusion criteria

UK based websites were included with information rich content on expressing breastmilk, pumping breastmilk and breast pumps. These were either dedicated websites on the topic of expressing or pumping breast milk or an area within a more general breastfeeding, pregnancy and childbirth maternity website. We therefore refer to “sites” when we are discussing both websites and areas within a more general website.

### Exclusion criteria

Non-UK websites, sites which were only marketing breast pumps and sites which had insufficient relevant information for in depth analysis.

### Selection of purposive sample

Two researchers (RM, AA) independently searched for websites which fitted our inclusion criteria and identified a range of sites which were categorised into three key groups of information sources about expressing breastmilk for UK women: commercial (including breast pump manufacturers), the NHS, and 3^rd^ sector organisations. These sites were then purposively sampled for analysis initially to provide two websites from each of the three types of information source. In addition sites for specific groups (ie premature infants; fathers) were added later to increase diversity. Triangulation was undertaken by exploring general pregnancy and birth sites identified by our search to ensure that key sources were not missed and to ensure accuracy of our interpretation. Sites were identified and data uploaded between July 2013 and September 2013. The search was updated in March 2014 (RM) to triangulate findings and search for disconfirming data. At this stage two additional sites (one NHS, one commercial) were included into our analysis. A sample size of ten sites was considered sufficient to cover the sites that women would be likely to access.

### Data extraction

The sites were initially reviewed by two researchers (RM, AA) to explore the site design and how information and messages were delivered. Data included the use of imagery (photos, drawings and videos), text, the use of language and tone (where audio files were used), music and links between website areas or to other sites.

Information from each site was read, observed, watched (video) or listened to (audio) and entered into NVivo (QSR NVivo version 10; QSR International, Doncaster, Australia) in different formats depending on how the information was presented on the website. The formats uploaded included: entire documents (usually pdf files), web text which was cut and pasted into a word document or as researcher descriptive and reflexive notes taken from observations of the content, design and structure of sites.

### Data analysis

A descriptive and thematic analysis was undertaken focussing on the site design, how messages were delivered and the use and placement of imagery and sound. This process was not predetermined and evolved from data in an iterative manner, with key themes emerging which were discussed between the researchers.

Text information sources uploaded to NVivo were coded thematically. Two sources were coded independently by the researchers (RM, AA) to develop the coding index. As agreement between the researchers was strong the remaining sources were coded by one researcher (RM) and the second researcher reviewed and discussed decisions (AA). The final interpretive thematic framework emerged through constant comparison of sites, coded data, researcher notes and discussion.

When presenting the results it was considered important to maintain our researcher independence in this commercially and politically sensitive area. We have therefore made every attempt to maintain anonymity of the sites and the quotations extracted. As a research team we were conscious of our professional backgrounds (midwifery and general practice), our experience in clinical practice in the NHS, our connections with the third sector and our role conducting infant feeding research. We aimed to be reflexive and as objectively critical as possible in relation to any potential conflicts of interest, which are declared at the end of this paper. PH’s role was to provide impartial comment on some of the issues that arose throughout the study. Ethics committee approval was not required, as the websites are publicly available.

## Results

### Sample characteristics

The characteristics of the purposive sample of ten information rich website providers for in-depth analysis are: commercial (n = 3); NHS (n = 3) and 3^rd^ sector organisations (n = 4). Three of the 3^rd^ Sector sites were registered charities and two of the ten sites (one NHS and one commercial) indicated that they adhered to principles/standards regarding the reliability of their health information. The search update in March 2014 highlighted the complexity and changeability of the landscape. Original websites were sometimes different, new websites had appeared or were more apparent in the search and discussion forums had continued to develop, Table 1.

**Table 1:** **Sample characteristics**

**Site type**	**Identified in September 2013**	**Identified in March 2014**
Commercial (C)	2	1*
NHS (N)	2	1*
3^rd^ Sector (TS)	4	

*indicates the site is within a general health or pregnancy website.

The site groupings are identified as follows: commercial sites are C1, C2 and C3; NHS sites are N1, N2 and N3 and 3^rd^ Sector sites are TS1, TS2, TS3 and TS4.

A variety of design techniques were adopted including text files, photographs or illustrations, PDF downloads, audio clips, video clips, and hypertext links. Most sites provided women with downloadable PDF files for further information and most of the NHS and 3^rd^ Sector websites had hypertext links to other related support organisations. The most sophisticated, colourful and complex sites were commercial websites while NHS sites tended to be more text based and had relatively few images. 3^rd^ Sector sites were more varied and one in particular (TS4) used more graphics such as photographs & video clips. Product placement including photographs/videos of different types of pumps and their use by attractive couples in clearly affluent surroundings or at smart office-based workplaces dominated the commercial sites. NHS and 3^rd^ Sector sites used images of women in more diverse environments. One commercial site (C2) had relatively few images of the mother with the baby but many images of the partner and other family members holding the baby which seemed to link with an overall message about the importance of involving others in baby feeding. Websites used the personal pronouns ‘you’ or ‘I’ to speak directly to the mother and in some instances the father of the infant. Some (C1 & 2, TS1) also used women’s stories as if the woman was speaking directly to other mothers.

### Marketing and advertising

Product advertisement and purchase options were overtly displayed on some sites while others used covert hypertext links to lead the reader to a purchasing opportunity such as feeding products, breastpumps, financial services, or other more general baby products. Commercial sites had a greater tendency to use hypertext links related to feeding problems as an opportunity to purchase feeding products and accessories such as gels, creams, nipple shields and breast pads. Two commercial sites (C1&2) linked to their own products and the third commercial site (C3), which was sponsored by a baby product company, had a range of adverts on each page including baby milk companies and breastpump manufactures. Two 3^rd^ Sector sites (TS2&3) had links to their own shopping areas (own or other products) or to other sites where mothers could purchase equipment for expressing, storing and feeding breastmilk. Marketing links were not always clear, for example a link which indicated information on hiring a pump led to a website offering opportunities to buy equipment. One UK 3^rd^ Sector site suggested that mothers could rent an electric pump, but led the mother to a USA based breastpump manufacturer’s website. Once linked through to breast pump purchase areas, pumps were generally exhibited in price order, most expensive pumps first, however this pattern was also apparent in one 3^rd^ Sector website (TS3).

None of the NHS sites contained commercial company marketing and one commercial site stated that it adhered to advertising placement principles ensuring that adverts were differentiated from health information.

### Thematic analysis

The three main themes arising from our analysis were: how expressing breastmilk is portrayed (3 sub themes); why women should express (4 sub themes); and recommendations (2 sub themes).

#### Theme 1: how expressing breastmilk is portrayed

The three subthemes in this topic are: expressing compared to breastfeeding; expressing as a skill that requires to be learned and the need for expressing to be discrete or hidden. Conflicting tensions between the website messages were identified. Using a breast pump was promoted as similar to breastfeeding but more convenient and at the same time meeting mothers’ requirement to be discrete, particularly in the three commercial sites. Further tension exists within and between sites that promote expressing as easy and also indicate it can be difficult or requires to be learned.

#### Expressing compared to breastfeeding

Breastfeeding was frequently used as the comparator for expressing breastmilk. For some sites this meant that expressing was promoted as being like breastfeeding but easier and more convenient. However, others identified expressing as less effective for milk extraction than breastfeeding.

The three commercial sites promoted expressing as being natural or like breastfeeding either through pump research where they had ‘*strived to understand nature and have become experts in breastfeeding over the years*’ (C2) or through pumps that ‘*gently mimic[s] your baby’s suckling*’ (C1). At the same time these sites (C1&2) and some non-commercial sites (TS3 & 4; N1) portrayed breastfeeding as ‘*not always easy at the outset*’ (C2) where ‘*mothers sometimes face challenges with breastfeeding*’ (TS4) or that ‘*it* [breastfeeding] *can be difficult and feel restricting*’ (TS3).

Expressing as being more convenient and flexible than breastfeeding was promoted by commercial websites (C1, C2) and one NHS resource (N1), with some suggestion that breastfeeding might be restrictive or time limiting, for example:

‘*and sometimes I’m not ready to feed or the timings gone wrong and there’s breastmilk in the fridge* [image of cup of breastmilk in fridge next to fresh fruits and vegetable] *and it’s always there for whenever he wants to feed*.’ (C1)

The above example promoting the ready availability of expressed breastmilk is comparable to how breastfeeding is promoted by avoiding the need to sterilise bottles, make-up and warm formula. However, the phrase ‘I’m not ready to feed’ implies expressed milk as having more immediate accessibility than feeding at the breast.

Expressing was described as comfortable, relaxing and easy only in relation to using a breastpump and only by commercial sites (C1, C”). For example :

‘*unique design [of pump] to let you sit back and express with comfort and ease – you don’t have to lean forward but can sit in a comfortable natural position***’** (C1).

It is unclear what this quote refers to regarding ‘leaning forward’; it might be other breast pumps or to breastfeeding. Women are often taught the principles of positioning and attachment when breastfeeding and often try multiple positions to enable successful breastfeeding. The images linked to the above quote suggested expressing could be achieved whilst in a more relaxed and comfortable posture.

Non-commercial sites (e.g. TS3, N1) were more likely to indicate that breastfeeding was more efficient for milk removal than expressing and/or that pumping can be a very different mechanical process from either breastfeeding or hand expressing.

‘*if your baby is separated from you […], it is important to mimic a baby’s sucking by hand expressing – a breast pump acts in a different manner*.’ (N1).

#### Expressing as a learned skill

Expressing, and hand-expressing in particular, was presented as a learned skill (C2,3; N1,3; TS4). Two sites (TS4, N3) stated that hand expressing was a technique ‘*all new mothers should learn*’ and another that it ‘*takes time to learn how to express by hand, but keep going*’ (C3). This site also indicated that ‘*not everyone finds breast pumps simple to use, particularly electric ones*.’ Perseverance was encouraged with an NHS site indicating that it would get easier over time.

‘*You can use hand expression or a pump – or both – to express your milk. Whichever method you use, you may find that practice helps*’ (N1).

This sense of perseverance was also conveyed in one woman’s story about how she ‘*struggled to build up small quantities at a time for the freezer stock*’ (TS1).

#### Expressing as discrete or hidden

The need to make expressing a covert act and to disguise expressing equipment was obvious only on the commercial sites and was clearly linked to marketing opportunities promoting bags and storage equipment to make ‘*expressing on the go more discrete’ (C1)* or pumps which were *‘small and discreet for convenient use at home or on the go’* (C2).

Women were also advised about ‘*storing milk in fridge discreetly’* (*C1*) again encouraging women to hide milk expression. Concealment was further emphasised in the commercial sites (C1&2) with offers of breastpads ‘*to avoid embarrassing stains’* (*C1*) and in relation to noise:

*‘I bought an electric pump. It was very noisy and I felt as if the whole office could hear me expressing! I then got a mini-electric pump which was also too loud! Embarrassed by the noise I got a manual breast pump which is very quiet. I don't feel self-conscious and it's as quick as the mini-electric pump.’ (C3)*

#### Theme 2: why women should express breastmilk

The subthemes around how sites justify their approach to expressing breastmilk and their focus are: expressing breastmilk and health; separation from baby (circumstances or choice); solving or preventing breastfeeding difficulties, and bonding and the relationship with the baby. All sites gave reasons why women should express their breastmilk although the emphasis differed. The overarching reason for expressing was to make sure the infant got the health benefits of breastmilk and this message was often threaded throughout the text of most websites.

#### Expressing breastmilk and health

The value of breastmilk for providing ‘*the best start’* (TS2) was emphasised with phrases such as ‘*the healthiest way’* (N2) or ‘*health-enhancing benefits of breast milk’* (C1) and this was also conveyed visually by for example showing expressed breastmilk stored in fridges packed with fresh fruit (C1). Several sites focused less directly on the health benefits of breastmilk partly because they were linked to or embedded in bigger parenting or breastfeeding websites which already emphasised the health benefits of breastfeeding, rather than solely of breastmilk (N1, N3, TS4). Some such sites only emphasised the health value of breastmilk for preterm infants, for example the importance of expressing as ‘*uniquely contributing to the wellbeing and development of her baby*’ (TS4: preterm infants subsection).

The relationship between breastmilk feeding and health was further demonstrated through involvement of health professionals, but this differed by site type. For example NHS sites tended to present a health professional as a source of knowledge and advice or occasionally to role model expressing (N3). Whereas commercial sites often used health professionals to endorse products or processes; for example, expressing to facilitate return to work: ‘*I felt very sad until my doctor introduced me to the world of breast pumps*’ (C2). It is interesting that a generic ‘doctor’ is chosen rather than a midwife and it could be for a variety of reasons. For example, this may reflect the age of the infant when mothers return to work and midwifery care has usually ceased, it may reflect the potentially international target audience for the website or it may be that the site developers believe doctor endorsement will be more persuasive to women.

#### Separation from the baby: circumstance or choice

Depending on which site was accessed expressing breastmilk was presented as either essential for new mothers or as a choice in relation to specific needs or circumstances. For example the NHS sites and three of the 3^rd^ Sector sites emphasised the need to provide breastmilk for sick or preterm infants:

‘*Your baby may be too small or too sick to begin breastfeeding, but you can still give them the best start by expressing your breast milk*’ [TS2]

Whereas separation between mother and infant was promoted as a bonus by two of the commercial websites:

’*this way your baby can continue to gain all the benefits of our breastmilk without you having to be there.*’ [C1].

Commercial sites encouraged the mother to have time out or be independent of her infant suggesting that expressing made feeding less restrictive. One site seemed to indicate that mothers could only go outside the home if she expressed her milk. For example a mother featured in a video clip gave one of the ‘*main benefits of using breastpump*’ as

‘*gives me a lot of freedom – I can pop to the shops, go for coffee with friends and leave my baby with her father knowing she is still getting my breastmilk as opposed to using formula*’ [C1].

Returning to work featured in several sites but not in those specifically for preterm infants or fathers. One 3^rd^ sector site (TS1) focused solely on returning to work or social events as a reason to express. NHS and 3^rd^ sector sites tended to provide practical information on expressing and storing milk at work often in connection with women’s employment rights, whereas the commercial sites frequently linked returning to work to offering a range of aesthetically pleasing products to enable this:

*‘[NAME OF BAG] makes expressing milk at work easy: It is small and compact, comes with a hands-free set and an elegant shoulder bag*.’ (C2)

Commercial sites were the only sites that mentioned sleep; for example *‘regular expressing allows you to catch up on sleep’* (C1) or less explicitly ‘*if you are tired and need your partner to take a turn with feeding your baby’* (C3).

#### Solving or preventing breastfeeding difficulties

All websites promoted expressing to help mothers manage breastfeeding difficulties, such as engorgement or difficulties latching. One NHS site and one commercial site suggested that expressing may help prevent problems such as blocked ducts or ‘*to help protect you against mastitis’* (C3). Expressing to increase milk supply or breastfeeding duration was promoted by all the commercial sites, one NHS site (N1) and a 3^rd^ sector site (TS4: preterm subsection):

‘*Expressing milk is also a great way to increase your milk supply’* (C3)

Information directed at mothers of non preterm infants also suggested mothers could ‘*encourage milk production by expressing your milk*’ (N1) and a stronger message indicated that expressing was required to ‘*establish and maintain milk supply*’ (C1) and provided a resource where ‘*our Healthcare Professional explains why expressing plays an invaluable role in supporting and prolonging breastfeeding’* (C1). One 3^rd^ sector site (TS4) promoted expressing as a tool to understand attachment and two sites (TS4 and C2) suggested that ‘*it can be really reassuring just to see your milk’* (TS4).

By contrast other sites (NHS and 3^rd^ sector) promoted expressing as temporary or as a potential solution to a difficult situation rather than being essential to breastfeeding:

*‘Sometimes, for a short period of time, you may be advised to express milk in order to increase milk production and get the baby back on track’* (N1)

#### Bonding and relationship with the baby

Expressing to enable bonding with the infant was only promoted by commercial sites e.g. to ‘*create wider family bond as other family can feed your baby’* and emphasised a need to routinely involve fathers in feeding:

*‘I wanted to help him experience that same closeness. I also wanted him to increase his confidence in taking care of Emma.’* (C2)

One commercial site acknowledged that fathers may feel *‘left out*’ and ‘*worried that you won't bond with your baby as well as your partner will*’ (C3), but also provided other solutions for the partner support (e.g. taking on more household chores, providing his partner with snacks while breastfeeding) and to be involved with the baby (e.g. playing games, taking her for a walk) before suggesting ‘*the possibility of you feeding your baby a bottle of expressed breastmilk*’ (C3). Expressing to involve the partner was given a more cautionary message on the other sites. For example, one NHS site cautioned waiting ‘*until your baby is a little older before regularly expressing milk for your partner to feed your baby,* (N2) and a 3^rd^ sector site spoke directly to fathers stating:

‘s*urprisingly, this* [feeding by the father] *may be less important than you expect if you are involved with your baby in other ways. But if you are still keen to feed your baby you could give expressed breastmilk’* (TS3).

#### Theme 3: Expressing rules and recommendations

Most sites were represented within this theme but with different emphasis and connections. Two recommendations that were common across the sites were: method (of expressing and feeding expressed milk) and timing (starting to express).

#### Recommendations on methods of expressing and feeding

Recommendations about how to express i.e. by hand or by pump were markedly different across the sites according to the particular purpose or philosophy of the website. For example two commercial sites (C1&2) only mentioned expressing by pump and also tended to emphasise the importance of owning a breastpump and this being essential for enabling or improving feeding:

*The [NAME] breast pumps are designed to help you give breast milk to your child for longer whilst making the whole experience easier and more comfortable’* (C1)

Hand-expressing was the main focus of the NHS and 3^rd^ Sector sites and although most gave information on both techniques they tended to use both overt and covert cues to indicate their preference for hand-expressing. For example NHS websites had several photographs or video clips demonstrating how to hand express but fewer breastpump images; one 3^rd^ Sector site (TS4) had two video clips on hand expressing (one for healthy term infants and one for preterm infants) but no breastpump images. Several sites recommend hand expressing as being easier, better and safer while using a pump was portrayed as more difficult; for example hand expressing described as the ‘*the simplest way of expressing your milk’* (TS2) or as having ‘*less risk of cross infection*’ (TS4). One 3^rd^ sector website (TS1) only provided information on hand expressing. Only one site (C3) provided broad information on all methods of expressing (hand, manual pump and electric pump) and this was the only site that explored potential costs and the practical application of the different expressing methods.

Information on how expressed milk should be fed to the infant lacked consistency; although there was an overall message that bottles might not be the best option. For example the three commercial sites advised against using bottles until ‘*breastfeeding is well established*’ and two NHS sites (N1, N3) favoured using a cup or spoon. Two sites indicated that a bottle was ‘more usual’ and gave information on the use of both cups and bottles (N1, TS3) another site also gave information on both cups and bottles but indicated a preference for cup feeding (TS1). Two sites (N2, TS4) made no mention of how the infant could be fed expressed milk. One site (N1), however, linked method of feeding preterm infants to the mother’s feeding choice:

‘*Expressed breast milk can be given to your baby by tube […], syringe or cup, and, later, if you don’t want to breastfeed, by bottle’*. (N1)

#### When to start?

Recommendations about when expressing should start were usually based on the age of the infant, length of breastfeeding or whether breastfeeding was already established. However, there was inconsistency and a lack of clarity in the recommendations; for example one commercial site (C1) suggested delaying expressing ‘*to allow breastfeeding to become established’* but mentioned many time points, for example: ‘*a few weeks’* or *‘four to six weeks after the birth*’ or ‘ *6–8 weeks of age*’ or ‘*at least three to six weeks of exclusive breastfeeding’* (C1)*.* Another commercial site (C3) advised the father to ‘*wait until your baby and your partner have got the hang of breastfeeding*.’ Non-commercial sites were equally vague about when to start expressing, one suggested ‘*It’s best to wait until your baby is a little older before regularly expressing milk’* then later*: ‘In the first few days it is easier to express by hand*’ (N2). Another didn’t give any clear guidance but talked about expressing ‘*in the early days’* (N1) and about expressing colostrum, implying the first 1–4 days. A 3^rd^ sector site stated that:

‘*expressing your breastmilk isn’t something you should feel you have to do straightaway – if at all. It’s a useful skill to have, but it’s usually easier if you wait until you’re confident in your milk supply and in your baby’s ability to breastfeed well*’ (TS3).

In a minority of sites the stage of breastfeeding or age of infant was linked to definite recommendations about which method of expressing to use:

‘*in the early days, when a mother needs to express, hand expressing will be far more successful than using a pump’* (TS4)

The advice on non-commercial sites for preterm or sick infants was more consistent suggesting mothers should ‘*express early – as soon as you can, and preferably within six hours of your baby’s birth*’ (N1) or ‘*within the first 4–6 hours*’ (TS4).

## Discussion

Our study illustrates how the portrayal of expressing breastmilk on websites is varied, often inconsistent and is likely to convey different meanings to parents. Each site exhibits individual preferences and purposes with the information it conveys, so that an individual would need to consult several sites to gain a complete and fully informed overview of this complex and skilled behaviour. Some sites had a clear bias towards specific methods of expressing and only three of the included ten sites provided information on both pumping and hand expressing. Information on using breastpumps is more readily available from commercial sites but was frequently embedded in text and imagery employing clear and persuasive marketing opportunities. Our analysis also found that the commercial sites emphasised some of the key concerns of new parents [19,20] as the rationale for expressing milk and for purchasing their equipment, such as sleep, shared feeding, the need for discretion and free time for mothers. In contrast, non-commercial sites portray a more theoretical rationale for the information and messages which they convey. Commercialisation of breast milk and breastfeeding is a concern expressed by others [10,21].

Messages around methods of expressing or feeding breastmilk in relation to the age of infant or duration breastfeeding (i.e. length of breastfeeding and whether breastfeeding was ‘established’) were mixed, sometimes contradictory and lacked clear justification. This may be explained by the paucity of evidence about how expressing breast milk impacts on feeding outcomes [13,14]. There is no clear evidence about the effects of expressing method, how expressed breastmilk is delivered, the age of infant/duration of breastfeeding when expressing commences, the frequency and intensity of expressing, or the proportion of feeds that are expressed on breastfeeding duration or the exclusivity in healthy term infants [13,14,22].

This is manifest in the inconsistency of UK policy and guidelines; for example the new Baby Friendly Initiative standards [23] recommend that mothers of healthy term infants should be taught and supported to hand express but only mention breast pumps in the context of the neonatal unit. By contrast the National Institute for Health and Clinical Excellence (NICE) postnatal guidelines [12], although advocating hand expressing, also suggest mothers should have access to breast pumps in hospital and be given instruction on their use. There is limited evidence to support these recommendations as most research focuses on pumping technique among mothers of sick or preterm rather than term infants [22,24-26]. A Cochrane review [13,22] included only two studies comparing pumping with hand expressing and/or breastfeeding among term healthy babies [27,28]. One study [27] demonstrated significantly more breastfeeding at 2 months among mothers who hand expressed compared to bilateral electric pumping. The other [28] compared the prolactin response from electric pumps, manual pumps and hand expressing with breastfeeding and noted that only the bilateral electric pump produced prolactin levels similar to or higher than breastfeeding and that hand expressing had a lower prolactin response and the lowest volume of milk.

We identified one cohort study [29] in which breast milk expression was positively associated with any breastfeeding at six months. In this study over 76% of participants were expressing in the first month the majority using a breast pump. No analysis was provided on effects of age of starting or method of expressing and we have been unable to identify any experimental research comparing the effect of the time of starting expressing i.e. early onset versus later onset or on the effect of method of expressing on breastfeeding duration. Therefore recommendations supporting hand expressing in the early weeks of life lack good evidence and may serve to discourage women from expressing at all since many women find hand expressing challenging [30]. In addition, women may experience negative feelings about the role of breasts and expressing milk and this can create challenges for how to negotiate both hand expressing and breastfeeding within social and cultural norms [31].

Some of the sites in our analysis seemed to conflate expressing with bottle feeding and advised against doing either until breastfeeding was established. This may relate to concerns about ‘nipple confusion’ if bottles are used for feeding expressed breastmilk [32], but this is not explicitly referred to and evidence for a negative impact of bottles on feeding outcomes is limited [33,34]. There is more robust evidence demonstrating shorter duration of breastfeeding among breastfed infants who also receive formula milk by bottle [4] but the causal pathway is uncertain. The effects of any expressing on breastfeeding duration among health term infants has therefore not been established [14].

Several publications have identified women’s concerns that their partner or other family members will not be able to bond with their infant if she is breastfeeding [19,20,35], in some cases women suggest that choosing to breastfeed is selfish [35] and some may feel uncomfortable when they perceive their partner to be excluded [20]. A partner’s lack of opportunity to bond may be perceived as a threat to family wellbeing [20] and to address this many women introduce formula milk or express their breastmilk [19,20,35]. Interestingly some of the commercial sites we reviewed use this as a reason to own/buy a breastpump. The persuasive arguments put forward in these sites accompanied by imagery showing partners and family members holding and feeding the infant could influence women’s desires to share feeding. Qualitative research with fathers of breastfed infants report their desire to experience feeding, with feeding being seen as a positive connection with the baby [19,20,35]. The sense of missing out on something special is in the context of several decades of promoting breastfeeding which has focused on bonding between mother and infant [36]. The notion of bonding through feeding is flawed on several levels: bonding has never been properly defined, the ‘critical’ time period for bonding has varied [37] and when originally conceptualised by Klaus & Kennel [38] did not solely relate to breastfeeding.

Access to breast pumps is a source of health and social inequalities [39]. Hand expressing is freely available to all women but commercial sites in our study promote breast pumps. Breast pumps range from £14.99 to £1374.30 on Amazon UK (date 12/11/2014). The more expensive pumps are large and electric, with some referred to as “hospital quality” inferring medical endorsement. The perceived need for or the desirability of breast pumps therefore has the potential to increase health inequalities for breastfeeding where inequalities are already evident. Breastfeeding duration and exclusivity are related to socioeconomic status with older and more affluent women breastfeeding for longer [4], and older and more affluent women are also more likely to express their breastmilk [9]. There is evidence that not being able to access (or afford) a breast pump results in women supplementing with artificial milk [40] and the loan of a free breast pump on return to work has been shown to delay formula supplementation [41]. However, if expressing by pump is not given as an option, as observed in several sites in our study, there is the risk that some women will use infant formula to support their lifestyle choices. This may be reinforced by marketing techniques which are clearly pitched at the more affluent women as suggested by imagery used on their sites. This could lead to further exclusion of mothers from lower socioeconomic backgrounds and therefore expanding the gap in social inequalities. In addition, providing a free breast pump was the most acceptable incentive strategy to support breastfeeding, in a UK survey of the general public [42].

### Strengths and limitations

To our knowledge this is the first study to compare and contrast website information which parents are likely to access to inform their decisions about expressing milk. This analysis comprises two historical snapshots of internet websites, with the updated search six months later finding rapid change in website availability, visibility and information. However, despite this historical limitation and the constant change of sites, we have identified important issues around conflicting advice and mixed messages resulting, we argue, from the lack of evidence in this area, which has opened up opportunities for commercial companies. Given the recent increases in breast milk expression, it is also possible that commercial companies have created opportunity by emphasising aspects of infant feeding that require women to express. This commercialisation could have adverse consequences for feeding outcomes, which could potentially exacerbate existing health inequalities. Our focus on UK relevant sites also limits the generalisablity to other countries and we identified a large number of North American sites, which UK women may access. This study started as a Masters of Health Research student project by AA and, due to resource constraints, parents were not involved in designing our search strategy therefore this may or may not reflect how they would search for information themselves. There is limited research on how individuals find health information but one paper [43] supports our approach indicating that users commonly enter short phrases into general search engines and few go beyond the first page of results. This reflects our search approach and indeed our sites all appear on the first or second page of Google. In addition, our study was informed by qualitative interviews with service users in two studies [15,39]. There is a need for further research to explore women’s perspectives on, and how they interact with websites and other information sources which might reveal new insights into expressing breast milk.

Several sites were part of larger sites on infant feeding or more general sites on pregnancy and parenting. For the purpose of this analysis we only explored the site area dedicated to expressing. Although this was sometimes alongside breastfeeding we did not explore all the content on infant feeding or breastfeeding. Therefore where we identified that some sites described expressing as a solution to breastfeeding difficulties, which could be interpreted as portraying breastfeeding either negatively or realistically [19], then this might offer some explanation. Our focus was on births of all gestation, and expressing for premature infants was only searched for specifically at the triangulation stage of the analysis.

## Conclusion

Our analysis of UK websites providing information about expressing breastmilk has identified inconsistent and incomplete information much of which is not grounded in research evidence. Individual websites often indicated preference for a particular method of expressing despite a lack of robust evidence showing any benefit. Furthermore many recommendations, such as delaying expressing until the infant has reached a particular age, were not supported by evidence. While the health benefits of breast milk are acknowledged across all the sites positive portrayal of breastfeeding appeared limited at times. Commercial sites in particular appear to focus their key reasons to express on concerns such as bonding, lack of sleep or the restrictions that breastfeeding may place on the mother. Although these concerns have been voiced in studies with parents the harm or benefit of expressing as a potential solution has not been explored. The lack of research evidence has opened opportunities for the commercialisation of breastfeeding which has the potential to further exacerbate health inequalities. In the context of changing infant feeding behaviour and parents’ expressed desire for reliable and consistent information on feeding their infant in a manner that fits their preferred lifestyles, access to good quality, holistic information is urgently required.
